# Associations Among Internet Addiction, Genetic Polymorphisms, Family Functioning, and Psychopathological Risk: Cross-Sectional Exploratory Study

**DOI:** 10.2196/17341

**Published:** 2020-12-24

**Authors:** Luca Cerniglia, Silvia Cimino, Eleonora Marzilli, Esterina Pascale, Renata Tambelli

**Affiliations:** 1 International Telematic University Uninettuno Rome Italy; 2 Sapienza - University of Rome Rome Italy

**Keywords:** internet addiction, mobile phones, family functioning, depression, anxiety, avoidant personality, MAO-A, 5-HTTPR, DRD4, DAT1

## Abstract

**Background:**

International research has emphasized that youths are at higher risk for the onset of internet addiction (IA), but studies investigating biological, psychological, and social factors associated with this condition are limited.

**Objective:**

This study aims to investigate the possible association between IA and genetic polymorphisms in monoamine oxidase A (MAO-A), serotonin-transporter (5-HTTPR), dopamine receptor (DRD4), and dopamine transporter (DAT1) genes by considering the role played by the perception of young adults in their family functioning and their depression, anxiety, and avoidant personality problems.

**Methods:**

In a sample of 104 male and female young adults aged between 19 and 23 years (mean age 21.87, SD 2.29 years) recruited from universities in the central southern part of Italy, we addressed the presence of IA using the Young criteria of the IA test. Moreover, the perception of young adults of their family functioning and their psychopathological symptoms were assessed through the Family Assessment Device (FAD) and the Adult Self-Report, respectively.

**Results:**

We found no significant association between IA and any genetic polymorphisms, neither among males or females. Young adults with IA reported significantly higher scores in the subscale of FAD affective responsiveness (AR; *P*=.01) and in depressive problems (*P*=.02), anxiety problems (*P*=.009), and avoidant personality problems (*P*=.003) than those in the control group. Results of mediation analyses showed a mediation role played by depressive symptoms (*B*=0.99; 95% CI 0.22 to 1.97) and avoidant personality problems (*B*=1.09; 95% CI 0.32 to 2.05) of young adults on the relationship between the FAD, AR, and IA. Finally, this relationship was moderated by the genotype of the 5-HTTLPR (*P*<.001), DAT1 (*P*<.001), and MAO-A (*P*<.001) genes in young adults.

**Conclusions:**

This exploratory study supports the recent evidence on the mutual relationship among biological, individual, and social risk factors associated with IA in young adulthood. Our findings may have important clinical implications for the development of prevention and treatment programs.

## Introduction

### Background

Over the past decade, the diffusion and use of the internet has grown rapidly, especially among adolescents and young adults [1]. From a developmental perspective, young adults may face important challenges in relationships with their family, peers, and society, the outcomes of which can be mediated and moderated by their psychological and biological characteristics [2]. On the one hand, youths can benefit from the digital revolution, which allows them to instantly search for information and communicate with others around the world [3]. In addition, there is evidence that mobile health smartphone apps can prevent diseases and improve the quality of life in young people [4]. However, on the other hand, scientific literature indicates that young adults are at higher risk of using the internet in a maladaptive way [5,6], to the point of developing internet addiction (IA) symptoms [7]. However, to date, official diagnostic criteria have not been identified [8]; although, the revision of the fifth edition of the Diagnostic and Statistical Manual of Mental Disorders (DSM-5) [9] has included an internet-related condition—the internet gaming disorder in Section III. This condition has been defined as a behavioral addiction that eventually leads to loss of control over internet gaming and functional impairment [10]. Some authors have defined IA as uncontrollable and obsessive-compulsive use of the internet, preoccupations regarding computer or technological devices, inability to control their use, poor time management, craving, and interpersonal problems [11-14]. Epidemiological studies have shown a prevalence of IA ranging from 6% to 35% among young adults [15,16] and from 6% to 21% among adolescents [17]. Although the highest prevalence has been reported among males [18], recent evidence has underlined an increase among females, with no significant gender difference [19]. The Developmental Psychopathology theoretical framework [20] offers a valid model to conceptualize clinical and subclinical psychological difficulties in young adulthood (such as IA) as it considers the development as a result of mutual influences between individual inherited genetic vulnerabilities and the quality of social experiences [21,22], particularly within the family context [23].

### Genetic Influences on IA

Genes involved in dopaminergic and serotoninergic systems are the candidate genes most frequently associated with IA [24]. Dopamine (DA) is an important monoamine that regulates various neural mechanisms, such as cognitive memory and emotional activity [25], and represents the final pathway of the reward system [26]. The availability of DA is regulated primarily by its transporter (DAT), which recaps dopamine at the level of nerve synapses [27]. The 3' untranslated region of the DAT1 gene contains a variable number of tandem repetitions (VNTRs) of 40 polymorphic base pairs, and the most frequent polymorphisms are 9 or 10 repetitions [28]. Neurobiological studies have shown a dysregulated dopamine transmission associated with IA [29,30], with a decreased level of expression of DAT in the striatum [31]. Another gene crucial to the dopaminergic system is the dopamine D4 receptor gene (DRD4), which is involved in a wide range of behavioral processes related to addiction, such as attention, motivation, and emotion [32]. This gene has a polymorphic 48-base pair VNTR in exon 3, and the most frequent polymorphisms are 4 (4R), 7 (7R), and 2 (2R) repeats [33]. Previous studies have underlined significant associations between the DRD4 4R genotype with increased attentiveness [34,35] that, in turn, has been associated with a higher risk of IA [36]. Besides the role played by dopamine, it has been evidenced that serotonin may also be implicated in IA. Serotonin (5-HT) plays an important role in feelings of well-being, happiness, anxiety, stress susceptibility [37], and depression [38]. The serotonin-transporter-linked polymorphic region (5-HTTLPR) is crucial for the fine regulation of 5-HT [39]. This functional polymorphism results in 2 common alleles, the short (S) and the long (L) [40], and studies by Sun et al [36] and Lee et al [41] have reported significant association between IA and the S/S genotype. Other studies involving allelic variants in monoamine oxidase A (MAO-A) activity have provided further support for the role played by serotonin and dopamine, given that this gene encodes an enzyme involved in the degradation of DA and 5-HT. The MAO-A gene has a 30-base pair repeat in the promoter region that affects transcriptional efficiency [42]. The high-activity variant (MAOA-H; 3.5 or 4 repeats) has shown significant association with impulsive personality traits [43] and tobacco and cannabis use [44], but other studies have reported conflicting results, with the low-activity variant (MAOA-L; 2, 3, or 5 repeats) associated with disordered gambling [45,46]. However, considering the complexity of IA, to date there is a dearth of studies that have considered possible interactions between genetic vulnerability and the risk provided by the social environment [47], although recent evidence has highlighted the genetic moderation on the impact of the environment on other disorders and behaviors related to addiction [48].

### The Role of Family Functioning

In this field, the quality of family functioning has been suggested to be one of the main environmental risk factors for the onset and maintenance of IA among young adults [49,50]. In particular, many studies have shown that the lack of parental emotional support and connectedness, and the poor quality of the relationships with parents, have an important influence on the risk taking and addictive behaviors of their offspring [51-53], including IA [54-56]. Moreover, it has been posited that the poor quality of family functioning is associated with a wide range of psychopathological problems [57-59], which in turn may lead to a higher risk for the onset of IA [60].

### Individual Psychopathological Symptoms and IA

International research has widely demonstrated the comorbidity between IA and other psychological difficulties, both in terms of psychopathological symptoms and personality disorder [61,62]. Although several studies have shown that this relationship may be reciprocal and bidirectional [63,64], recent evidence has suggested that IA can be considered as the result of other psychopathological problems [20], resulting in a strategy to cope with psychological discomfort [2] and physical discomfort [65]. Depression and anxiety symptoms represent the 2 psychopathological areas most frequently associated with IA, both in clinical samples [66] and in the general population [67,68]. Moreover, significant associations with cluster C personality features have been evidenced [69], especially with traits of avoidant personality disorder [70].

### This Study

From a biopsychosocial perspective [71], this exploratory study aims to evaluate the role played by biological, psychological, and social factors that may contribute to the etiology of IA in a sample of 104 young adults of the general population.

We hypothesized the presence of the following: (1) significant associations between IA and genetic polymorphisms in MAO-A (ie, MAOA-H genotype), 5-HTTPR (ie, S/S genotype), DRD4 (ie, 4R/4R genotype), and DAT1 (ie, 9-repeat genotype) genes, based on previous studies that have underlined the key role of these genes in the biochemistry of addiction disorder [45,72,73], including IA [36,41,74,75]; (2) significant differences in the quality of family functioning of young adults and psychological profiles (ie, depression and anxiety problems, avoidant personality problems) between youths with IA and the control group, as suggested by previous studies that have shown significant association between IA both with a poor quality of family functioning [49,50] and with psychopathological symptoms, especially in the areas of depression and anxiety symptoms [76,77] and traits of avoidant personality disorder [78]; (3) a mediation role played by the psychological profile of young adults on the relationship between family functioning and IA, based on studies that have shown a predictive effect of the quality of family functioning on the psychopathological profile of young adults [57,58] that, in turn, has been suggested to be a significant predictor of IA [70]; and (4) the moderator role played by MAO-A, 5-HTTPR, DRD4, and DAT1 genotypes on the relationship between family functioning and IA, as suggested by recent evidence that has underlined that individual genotype play an important role in moderating individual sensitivity to environmental events [79,80] and to the development of psychopathology related to addiction [48].

## Methods

### Recruitment

Over a period of 1 year, 150 young adults (65/150, 43.3% boys; 86/150, 56.7% girls) aged between 19 and 23 years were recruited for this study, through the collaboration of universities in the center-south of Italy. All youths signed informed consent, in which the study was illustrated in detail. To examine the youths’ genotype of the MAO-A, DAT1, 5-HTTPR, and DRD4 genes, we collected biological materials from human buccal swabs (the procedure is described below). In addition, the youths who agreed to participate in this study were administered self-report questionnaires (described below). This study was approved by the Ethical Committee of the Department of Dynamic and Clinical Psychology at Sapienza University of Rome, in accordance with the Declaration of Helsinki.

### Procedure for Biological Sampling

Youths were assessed through buccal swabs (Isohelix Swab Pack) by a group of psychologists, specifically trained for the purposes of the study. Subjects were aware of not having to eat (including chewing gum, candy, etc), drink (except water), smoke, and brush their teeth for at least 1 hour before sampling. Epithelial cell samples were carefully collected through buccal swabs. The biological samplings were transported, slightly chilled by Normative ice (+4°C), to the laboratories of the co-author, EP, for further processing. After buccal swabs were gathered, young adults filled self-report questionnaires (described below).

### Methods

#### Assessment of Internet Use or Abuse Among Young Adults

The IA test (IAT) [81] is a 20-item, 5-point Likert scale that measures the severity of self-reported compulsive use of the internet. Total IA scores were calculated, with possible scores ranging from 20 to 100. The scale showed very good internal consistency, with a Cronbach α value of .82 in this study. According to Italian validation [82], total IAT from 0 to 39 represents average users with complete control of their internet use, scores from 40 to 69 represent excessive internet use, and scores from 70 to 100 represent significant problems because of internet use.

#### Assessment of Psychological Profiles of Young Adults

The Adult Self-Report (ASR) [83] is a self-report used to elicit information regarding psychological functioning. Items are assessed on a 3-point Likert scale (0=not true, 1=sometimes true, and 2=very often true). The aim of this study is to explore the role played by depression, anxiety, and avoidant personality problems of young adults on IA. Consequently, we used the scores of the following DSM-oriented scales: depressive problems, anxiety problems, and avoidant personality problems. Research has demonstrated good reliability and validity for the scales of the ASR [83]. In this study, the ASR showed good internal coherence (Cronbach αalpha=.73-.84).

#### Assessment of Family Functioning of Young Adults

The Family Assessment Device (FAD) [84,85] is a self-report questionnaire that was developed to measure perceptions of family functioning. It is composed of 60 items evaluated on a 4-point scale (1=strongly agree and 4=strongly disagree), and measures the 6 dimensions of the McMaster Model of Family Functioning: (1) problem solving, which refers to the family’s ability to solve problems; (2) communication, which refers to whether communication in the family is clear and direct or vague and indirect; (3) roles, which addresses the issue of how roles and responsibilities are allocated among family members; (4) affective responsiveness (AR), which refers to the ability of the family members to respond to a range of situations with appropriate quality and amount of emotion; (5) affective involvement refers to how family members experience interest in and involvement with each other; and (6) behavioral control, which assesses whether the family has norms or standards governing individual behavior and responses to emergency situations. In all dimensions, higher scores represent less satisfaction with family functioning. The validity, reliability, and internal consistency of individual scales have been thoroughly investigated [86]. The internal consistency of the 6 subscales in this study was also adequate (Cronbach α=.77-.87).

#### DNA Isolation and Genotyping

Buccal cell DNA isolations were performed using the Buccal Prep Plus DNA isolation kit (Isohelix) according to the manufacturer’s instructions. The yield of DNA is usually between 3 and 10 µg. Allelic variants of the MAO-A, DAT1, 5-HTTPR, and DRD4 genes were identified. Genotypes were grouped into dominant, recessive, and additive genetic models, with the exception of MAO-A among males. The MAO-A gene is located on the X chromosome, so males have 1 allelic variant. Consequently, for males, the MAO-A gene was grouped based on the presence of the MAOA-H activity allele (ie, 4R) and the MAOA-L activity alleles (ie, 2R, 3R, and 5R). For females, the MAO-A gene was grouped based on the presence of 2 copies of the MAO-H allele, at least one copy of the MAO-H allele or 2 copies of the MAO-L allele. For the DRD4 gene, previous studies [87,88] have emphasized that the 4R allele is the ancestral allele and has distinct functionality with regard to other alleles. Consequently, subjects were grouped based on the presence of 2 copies of the 4R allele, at least one copy of the 4R allele, and without the 4R allele. With regard to the 5-HTTPR genotype, we considered the presence of 2 copies of the S allele (ie, S/S), at least one copy of the S allele (ie, S/L), and 2 copies of the L allele (ie, L/L). Finally, for the DAT1 gene, groups were formed based on the presence of 2 copies of the 10R allele (ie, 10/10), at least one copy of the 10R allele (ie, 9/10), and 2 copies of the 9R allele.

#### Statistical Analysis

Preliminary analyses were performed using descriptive statistics (frequencies, percentages, and mean scores). Deviations from Hardy-Weinberg equilibrium were tested using the chi-square test, which allows the comparison of the expected frequency of the specific polymorphism with the observed one [89]. The Hardy-Weinberg equilibrium of the MAO-A among males was tested using the method proposed by Graffelman and Weir [90] for biallelic variants on the X chromosome, which considers both males and females. To verify the possible association between IA (IA group vs control group) and genetic polymorphisms in MAO-A, 5-HTTPR, DRD4, and DAT1 genes, considering the role played by gender, sex-stratified logistic-regression models were conducted. For each marker, we examined the dominant, recessive, and additive genetic models. An allelic model was used to examine the possible effect of MAO-A among males. Odds ratios and 95% CIs are presented. To verify the possible differences between the 2 groups on all the FAD subscales and on the ASR scores of the DSM-oriented subscales of depressive problems, anxiety problems, and avoidant personality problems, two tailed *t* test for independent samples was carried out. Based on preliminary analyses, a total sample bootstrap mediation analysis for simple and multiple mediation was then conducted to explore the possible mediation effects of the emotional-behavioral functioning of young adults on the relationship between the perception of young adults of their family functioning and their scores on IAT. The scores of the independent variable and of the mediators were standardized before performing the mediation analyses. Indirect (ie, mediating) effects were evaluated with 95% bias-corrected CI based on 5000 bootstrap samples. Finally, we tested whether the relationship between FAD and IAT could be moderated by genetic polymorphisms in the MAO-A, 5-HTTPR, DRD4, and DAT1 genes in young adults. For moderation analyses, only dominant genetic models were used. We standardized the score of the independent variable before performing the moderation analyses. All analyses were performed using IBM SPSS software 25.0. Mediation and moderation analyses were performed using PROCESS for SPSS [91].

## Results

### Sample Characteristics

In this study, from the total sample of 150 youths, 16.6% (25/150) of youths who did not complete the assessment procedure and 12.6% (19/150) of youths for whom it was not possible to collect biological samples were excluded. The final sample consisted of 104 young adults (mean age 21.87, SD 2.29 years; 50/104, 48.1% males). All youths recruited were university students. Most of the youths recruited for the study lived in families (101/104, 97.1%). Of the parents, 88.5% (92/104) were married or cohabiting, whereas 11.5% (12/104) were separated. On the basis of the IAT cut-off for Italian validation (≥40) [82], for aims 1 and 2 of this study, the total sample was divided into 2 subgroups: (1) IA group: comprising youths who reported excessive internet use (35/104, 33.6%) and (2) control group: comprising youths in complete control of their internet use (69/104, 66.4%).

The allele and genotype frequencies of the DRD4, MAO-A, 5-HTTPR, and DAT1 genes among males (Table 1) and females (Table 2) are reported in this study.

Genotypes were distributed according to the Hardy-Weinberg equilibrium among male (DRD4: *χ^2^_1_*=2.1, *P*=.14; 5HTTPR: *χ^2^_1_*=.07, *P*=.78; DAT1: *χ^2^*_1_=1.2, *P*=.26) and female (DRD4: *χ^2^_1_*=.04, *P*=.83; 5HTTPR: *χ^2^_1_*=.1, *P*=.65; DAT1: *χ^2^_1_*=.4, *P*=.48) youths, with the exception of MAO-A (overall: *χ^2^_3_*=7.8, *P*=.05; females: *χ^2^_3_*=7.1*, P*=.007).

**Table 1:** Allele and genotype frequencies of the dopamine D4 receptor, monoamine oxidase A, serotonin-transporter, and dopamine active transporter 1 genes among males.

Genes			DRD4^a^, n (%)		MAO-A^b^, n (%)		5-HTTLPR^c^, n (%)		DAT1^d^, n (%)
**Allele**									
	2	5 (5)		0 (0)		N/A^e^		N/A	
	3	3 (3)		16 (32)		N/A		N/A	
	4	72 (72)		33 (66)		N/A		N/A	
	5	3 (3)		1 (2)		N/A		N/A	
	6	1 (1)		N/A		N/A		N/A	
	7	5 (5)		N/A		N/A		N/A	
	8	4 (4)		N/A		N/A		N/A	
	L	N/A		N/A		48 (48)		N/A	
	S	N/A		N/A		52 (52)		N/A	
	9	N/A		N/A		N/A		32 (32)	
	10	N/A		N/A		N/A		62 (62)	
**Genotype**									
	2R/2R	1 (2)		N/A		N/A		N/A	
	2R/4R	8 (16)		N/A		N/A		N/A	
	2R/5R	1 (2)		N/A		N/A		N/A	
	2R/7R	1 (2)		N/A		N/A		N/A	
	3R/3R	0 (0)		N/A		N/A		N/A	
	3R/4R	2 (4)		N/A		N/A		N/A	
	3R/7R	0 (0)		N/A		N/A		N/A	
	3R/8R	1 (2)		N/A		N/A		N/A	
	4R/4R	28 (56)		N/A		N/A		N/A	
	4R/5R	2 (4)		N/A		N/A		N/A	
	4R/7R	2 (2)		N/A		N/A		N/A	
	4R/8R	3 (6)		N/A		N/A		N/A	
	5R/7R	0 (0)		N/A		N/A		N/A	
	6R/7R	1 (2)		N/A		N/A		N/A	
	7R/7R	1 (2)		N/A		N/A		N/A	
	L/L	N/A		N/A		12 (24)		N/A	
	L/S	N/A		N/A		24 (48)		N/A	
	S/S	N/A		N/A		14 (28)		N/A	
	9/9	N/A		N/A		N/A		5 (12)	
	9/10	N/A		N/A		N/A		27 (42)	
	10/10	N/A		N/A		N/A		18 (36)	

^a^DRD4: dopamine D4 receptor gene.

^b^MAO-A: monoamine oxidase A.

^c^5-HTTLPR: serotonin-transporter-linked polymorphic region.

^d^DAT1: dopamine transporter 1.

^e^N/A: not applicable.

**Table 2:** Allele and genotype frequencies of the dopamine D4 receptor, monoamine oxidase A, serotonin-transporter, and dopamine active transporter 1 genes among females.

Genes		DRD4^a^, n (%)	MAO-A^b^, n (%)	5-HTTLPR^c^, n (%)	DAT1^d^, n (%)
**Allele**					
	2	7 (6.5)	2 (1.8)	N/A^e^	N/A
	3	11 (10.2)	33 (30.6)	N/A	N/A
	4	84 (77.7)	68 (63)	N/A	N/A
	5	3 (2.7)	5 (4.6)	N/A	N/A
	6	0 (0)	N/A	N/A	N/A
	7	3 (2.7)	N/A	N/A	N/A
	8	0 (0)	N/A	N/A	N/A
	L	N/A	N/A	62 (57.4)	N/A
	S	N/A	N/A	46 (42.6)	N/A
	9	N/A	N/A	N/A	42 (38.9)
	10	N/A	N/A	N/A	66 (61.1)
**Genotype**					
	2R/2R	1 (1.9)	1 (1.9)	N/A	N/A
	2R/4R	5 (9.3)	N/A	N/A	N/A
	2R/5R	0 (0)	N/A	N/A	N/A
	2R/7R	0 (0)	N/A	N/A	N/A
	3R/3R	3 (5.6)	9 (16.7)	N/A	N/A
	3R/4R	3 (5.6)	15 (27.8)	N/A	N/A
	3R/7R	2 (3.7)	N/A	N/A	N/A
	3R/8R	0 (0)	N/A	N/A	N/A
	4R/4R	37 (68.5)	26 (48.1)	N/A	N/A
	4R/5R	2 (3.7)	1 (1.9)	N/A	N/A
	4R/7R	0 (0)	N/A	N/A	N/A
	4R/8R	0 (0)	N/A	N/A	N/A
	5R/5R	N/A	2 (3.7)	N/A	N/A
	5R/7R	1 (1.9)	N/A	N/A	N/A
	6R/7R	0 (0)	N/A	N/A	N/A
	7R/7R	0 (0)	N/A	N/A	N/A
	L/L	N/A	N/A	17 (31.5)	N/A
	L/S	N/A	N/A	28 (51.9)	N/A
	S/S	N/A	N/A	9 (16.7)	N/A
	9/9	N/A	N/A	N/A	9 (16.6)
	9/10	N/A	N/A	N/A	23 (42.6)
	10/10	N/A	N/A	N/A	22 (40.8)

^a^DRD4: dopamine D4 receptor gene.

^b^MAO-A: monoamine oxidase A.

^c^5-HTTLPR: serotonin-transporter-linked polymorphic region.

^d^DAT1: dopamine transporter 1.

^e^N/A: not applicable.

### Association Between IA and Genetic Polymorphisms

To verify the possible association between IA and genetic polymorphisms in MAO-A, 5-HTTPR, DRD4, and DAT1 genes among male and female youths, sex-stratified logistic-regression models were conducted. If the CI crosses 1, the difference between genotype groups can be considered not significant. As shown in Tables 3 and 4, no significant associations were found between IA and any considered genotypes, both among males and females (all CIs crossed 1).

**Table 3:** Association between internet addiction and genetic polymorphisms in serotonin-transporter, dopamine active transporter 1, dopamine D4 receptor, and monoamine oxidase A among male youths.

Gene and genotype		Wald χ^2^ (*df*)	*P* value	OR^a^ (95% CI)
**5-HTTPR^b^**				
	S/S vs S/L+L/L^c^	0.5 (1)	.47	1.57 (0.45-5.45)
	L/L vs S/S+S/L^d^	0.001 (1)	.97	0.98 (0.26-3.66)
	S/S^e^	0.5 (2)	.75	N/A^f^
	S/L	0.05 (1)	.67	1.4 (0.29-6.62)
	L/L	0.3 (1)	.80	0.84 (0.20-3.45)
**DAT1^g^**				
	10/10 vs 9/10+9/9^c^	0.1 (1)	.73	0.81 (0.25-2.65)
	9/9 vs 9/10+10/10^d^	0.7 (1)	.39	2.25 (0.34-14.83)
	10/10^e^	0.7 (2)	.69	N/A
	9/10	0.6 (1)	.40	0.42 (0.05-3.21)
	9/9	0.6 (1)	.43	0.45 (0.06-3.21)
**DRD4^h^**				
	4/4 vs non 4/4^c^	0.5 (1)	.47	0.65 (0.21-2.06)
**MAO-A^i^**				
	H vs L^j^	0.007 (1)	.93	0.95 (0.29-3.11)
	L vs H	0.007(1)	.93	1.05 (0.32-3.45)

^a^OR: odds ratio.

^b^5-HTTLPR: serotonin-transporter-linked polymorphic region.

^c^Dominant.

^d^Recessive.

^e^Additive.

^f^ N/A: not applicable.

^g^DAT1: dopamine transporter 1.

^h^DRD4: dopamine D4 receptor gene.

^i^MAO-A: monoamine oxidase A.

^j^Allelic.

**Table 4:** Association between internet addiction and genetic polymorphisms in serotonin-transporter, dopamine active transporter 1, dopamine D4 receptor, and monoamine oxidase A among female youths.

Gene and genotype		Wald *χ*^2^ (*df*)	*P* value	OR^a^ (95% CI)
**5-HTTPR^b^**				
	S/S vs S/L+L/L^c^	0.07 (1)	.78	0.78 (0.14-4.32)
	L/L vs S/S+S/L^d^	0.1 (1)	.69	1.29 (0.35-4.68)
	S/S^e^	0.1 (2)	.91	N/A^f^
	S/L	0.1 (1)	.69	0.68 (0.10-4.52)
	L/L	0.1 (1)	.74	0.80 (0.20-3.08)
**DAT1^g^**				
	10/10 vs 9/10+9/9^c^	1.1 (1)	.28	0.48(0.13-1.82)
	9/9 vs 9/10+10/10^d^	0.0 (1)	.78	0.78 (0.14-4.32)
	10/10^e^	1.6 (2)	.43	N/A
	9/10	0.06 (1)	.79	0.77 (0.11-5.24)
	9/9	0.4 (1)	.49	1.86 (0.31-11.18)
**DRD4^h^**				
	4/4 vs non 4/4^c^	0.4 (1)	.48	1.55 (0.44-5.42)
**MAO-A^i^**				
	H/H vs H/L+L/L^c^	0.2 (1)	.64	0.75 (0.22-2.55)
	L/L vs H/H+H/L^d^	0.6 (1)	.41	0.50 (0.09-2.62)
	H/H^e^	1.7 (2)	.42	N/A
	H/L	0.2 (1)	.65	0.66 (0.11-3.91)
	L/L	0.9 (1)	.31	2 (0.51-7.81)

^a^OR: odds ratio.

^b^5-HTTLPR: serotonin-transporter-linked polymorphic region.

^c^Dominant.

^d^Recessive.

^e^Additive.

^f^ N/A: not applicable.

^g^DAT1: dopamine transporter 1.

^h^DRD4: dopamine D4 receptor gene.

^i^MAO-A: monoamine oxidase A.

### IA, Family Functioning, and Psychopathological Symptoms of Young Adults

To verify possible differences between the 2 groups in family functioning and the psychological profile of young adults (ie, depression, anxiety, and avoidant personality problems), an independent samples *t* test was conducted. Results showed significant differences between youths with IA and their peers in the control group in the scores of the FAD AR subscale scores and ASR scores in all psychopathological areas (Table 5).

**Table 5:** t test for family functioning and psychological profile for the 2 groups.

Groups or study variables		IAG^a^ (n=35), mean (SD)	CG^b^ (n=69), mean (SD)	*t* test (*df*)	*P* value
**FAD^c^**					
	PS^d^	13.60 (2.95)	12.98 (3.36)	0.91 (102)	.36
	CM^e^	20.31 (2.97)	19.36 (2.69)	1.64 (102)	.10
	RL^f^	25.97 (2.13)	26,27 (2.02)	–0.71 (102)	.47
	AR^g^	15.25 (2.47)	13.95 (2.62)	2.43 (102)	.01
	AI^h^	18.25 (2.01)	18.59 (1.44)	–0.97 (102)	.33
	BC^i^	21.51 (2.09)	20.73 (2.48)	1.58 (102)	.11
**ASR^j^**					
	Depression	7.80 (5.08)	5.66 (4.16)	2.28 (102)	.02
	Anxiety	7.25 (2.59)	5.94 (2.24)	2.68 (102)	.009
	Avoidant personality	4.94 (2.85)	3.27 (1.90)	3.11 (102)	.003

^a^IAG: internet addiction group.

^b^CG: control group.

^c^FAD: Family Assessment Device.

^d^PS: problem solving.

^e^CM: communication.

^f^RL: roles.

^g^AR: affective responsiveness.

^h^AI: affective involvement.

^i^BC: behavioral control.

^j^ASR: Adult Self-Report.

### The Mediation Role Played by the Psychological Profile on the Relationship Between Family Functioning and IA

**Figure 1:**
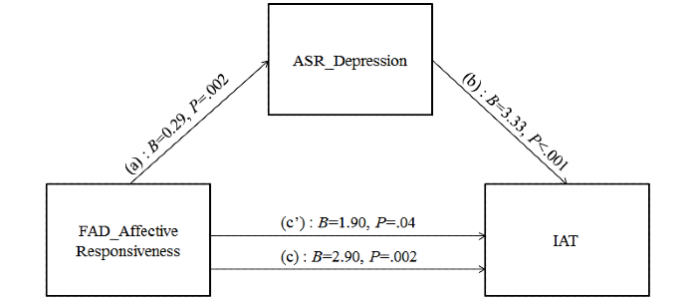
Simple mediation model for depressive problems mediating the association between affective responsiveness and internet addiction. (a) Direct effect of affective responsiveness on depression; (b) direct effect of depression on internet addiction test (IAT) score; (c’) direct effect of affective responsiveness on IAT score; (c) total effect (a*b+c’) of affective responsiveness on IAT score. ASR: Adult Self-Report; FAD: Family Assessment Device; IAT: internet addiction test.

On the basis of the previous analyses, we verified the possible simple and multiple mediation effects of depressive and anxiety symptoms and avoidant personality traits on the relationship between FAD AR and IA. Mediation analyses were performed using the SPSS Macro PROCESS [91], which provides coefficient estimates for the total, direct, and indirect effects of variables using ordinary least squares regression. In particular, Model 4 [91] was used, which allows us to estimate the indirect effects within a 95% CI. The indirect effect can be considered statistically significant if the CI does not include zero. In our first simple mediation model, we tested whether youth’s depressive symptoms mediated the effect of FAD AR on youth’s IA. Results showed that FAD AR was significantly associated with youth depressive symptoms (*B*=0.29; *P*=.002), which, in turn, was positively related to the youth’s scores on IAT (*B*=3.33; *P*<.001). Moreover, considering the relationship between FAD AR and IAT, both direct (*B*=1.90; *P*=.04) and total effect (*B*=2.90; *P*=.002) were significant (Figure 1).

With regard to the indirect effect, the bootstrap CI showed that the indirect path via depression was statistically significant (Table 6).

**Figure 2:**
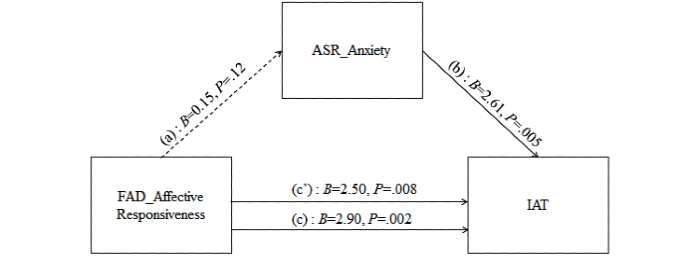
Simple mediation model for anxiety problems mediating the association between affective responsiveness and internet addiction. (a) Direct effect of affective responsiveness on anxiety problems; (b) direct effect of anxiety problems on IAT score; (c’) direct effect of affective responsiveness on IAT score; (c) total effect (a*b+c’) of affective responsiveness on IAT score. ASR: Adult Self-Report; FAD: Family Assessment Device; IAT: internet addiction test.

We then tested whether the anxiety symptoms of young adults mediated the relationships between the FAD AR and IA. The results showed that the indirect effect of FAD AR through anxiety symptoms of young adults was not significant (Table 6). However, both direct (*B*=2.50; *P*=.008) and total effect (*B*=2.90; *P*=.002) of FAD AR on IAT were significant. Moreover, anxiety problems of young adults were significantly associated with their scores on IAT (*B*=2.61; *P*=.005), but the relationship between FAD AR and depression in young adults was not significant (*P*=.12; Figure 2).

**Figure 3:**
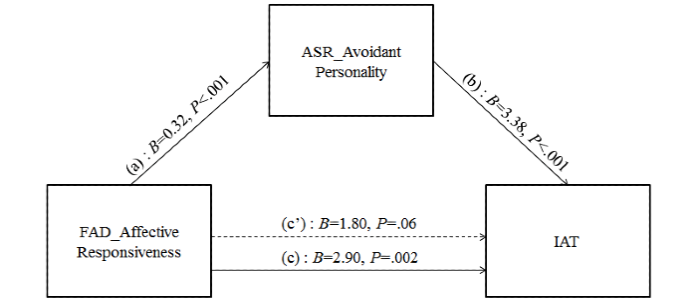
Simple mediation model for avoidant personality problems mediating the association between affective responsiveness and internet addiction. (a) Direct effect of affective responsiveness on avoidant personality traits; (b) direct effect of avoidant personality traits on IAT score; (c’) direct effect of affective responsiveness on IAT score; (c) total effect (a*b+c’) of affective responsiveness on IAT score. ASR: Adult Self-Report; FAD: Family Assessment Device; IAT: internet addiction test.

In relation to the possible mediation role played by avoidant personality traits of young adults on the relationship between FAD AR and IA, results showed that FAD AR was significantly related to their avoidant personality traits (*B*=0.32; *P*<.001) that, in turn, was significantly associated with the IA of young adults (*B*=3.38; *P*<.001; Figure 3).

**Table 6:** Simple mediator models showing total, direct, and indirect effects of affective responsiveness on internet addiction.

Psychopathological areas and paths			Path effect	
			B (SE)	95% CI
**Depression**				
	**Direct path**			
		AfRes^a^→DEP^b^	0.29 (0.09)	0.10 to 0.48
		DEP→IAT^c^	3.33 (0.94)	1.46 to 5.20
		AfRes→IAT	1.90 (0.94)	0.04 to 3.77
	**Indirect path**			
		AfRes→DEP→IAT	0.99 (0.44^d^)	0.22 to 1.97^e^
	**Total^f^**			
		AfRes→IAT	2.90 (0.94)	1.01 to 4.78
**Anxiety**				
	**Direct path**			
		AfRes→ANX^g^	0.15 (0.09)	−0.04 to 0.34
		ANX→IAT	2.61 (0.92)	0.77 to 4.46
		AfRes→IAT	2.50 (0.92)	0.66 to 4.34
	**Indirect path**			
		AfRes→ANX→IAT	0.39 (0.35^d^)	−0.11 to 1.24^e^
	**Total^h^**			
		AfRes→IAT	2.90 (0.94)	1.01 to 4.78
**Avoidant personality**				
	**Direct path**			
		AfRes→AvP^i^	0.32 (0.09)	0.13 to 0.50
		AvP→IAT	3.38 (0.94)	1.49 to 5.26
		AfRes→IAT	1.80 (0.94)	−0.07 to 3.69
	**Indirect path**			
		AfRes→AvP→IAT	1.09 (0.45^d^)	0.32 to 2.05^e^
	**Total^j^**			
		AfRes→IAT	2.90 (0.94)	1.01 to 4.78

^a^AfRes: affective responsiveness.

^b^DEP: Depression.

^c^IAT: internet addiction test.

^d^Bootstrapped SE values.

^e^Bootstrap CI values.

^f^Proportion mediated by indirect effect is 0.34%.

^g^ANX: Anxiety.

^h^Proportion mediated by indirect effect is 0.13%.

^i^AvP: avoidant personality.

^j^Proportion mediated by indirect effect is 0.37%.

The indirect path via avoided personality traits of young adults was also statistically significant (Table 6).

Finally, results of the multiple mediation analyses showed a significant total indirect effect (*B*=1.24; 95% bootstrapped CI 0.28-2.38), implying that the changes in ASR depression, anxiety, and avoidant personality collectively mediated the relationship between FAD AR and IA. However, the specific indirect effects of the 3 mediators were not significant (CI included zero; Table 7).

**Table 7:** Multiple mediator model showing total, direct, and indirect effects of affective responsiveness on internet addiction.

Paths		Path effect	
		B (SE)	95% CI
**Direct path**			
	(A_1_)AfRes^a^→DEP^b^	0.29 (0.09)	0.10 to 0.48
	(A_2_)AfRes→ANX^c^	0.15 (0.09)	−0.04 to 0.34
	(A_3_)AfRes→AvP^d^	0.32 (0.09)	0.13 to 0.50
	(B_1_) DEP→IAT^e^	1.86 (1.23)	−0.59 to 4.32
	(B_2_) ANX→IAT	0.60 (1.16)	−1.69 to 2.91
	(B_3_) AvP→IAT	1.84 (1.29)	−0.73 to 4.42
	(C’)AfRes→IAT	1.65 (0.95)	−0.23 to 3.55
Indirect path (A*B)^f^			
	Total	1.24 (0.53)	0.28 to 2.38
	AfRes→DEP→IAT	0.55 (0.42)	−0.17 to 1.48
	AfRes→ANX→IAT	0.09 (0.26)	−0.33 to 0.76
	AfRes→AvP→IAT	0.59 (0.47)	−0.27 to 1.60
Total (C=A*B+C’)^g^			
	AfRes→IAT	2.90 (0.94)	1.01 to 4.78

^a^AfRes: affective responsiveness.

^b^DEP: depression.

^c^ANX: anxiety.

^d^AvP: avoidant personality.

^e^IAT: internet addiction test.

^f^Bootstrapped SE values and CI values.

^g^Proportion mediated by specific indirect effect of AfRes→DEP→IAT is 0.24%, AfRes→ANX→IAT is 0.05%, and AfRes→AvP→IAT is 0.26%.

### The Moderator Role Played by Polymorphisms in Young Adults on the Relationship Between Family Functioning and IA

**Figure 4:**
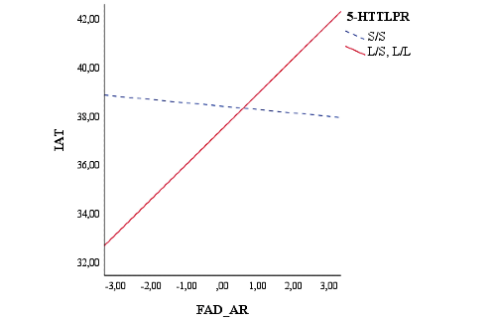
Moderation of youth’s 5-HTTLPR genotype on the relationship between the youth’s family functioning and IAT score. 5‐HTTLPR: Serotonin-transporter linked polymorphic region; FAD_AR: Family Assessment Device Affective Responsiveness; IAT: internet addiction test.

Finally, we evaluated the possible moderating role played by the genetic polymorphisms in young adults in MAO-A, 5HTTPR, DRD4, and DAT1 genes on the relationship between FAD AR and their scores on IAT. Moderation analyses were conducted using the method outlined by Hayes [91] (Model 1). The results showed that the relationship between FAD AR and IA was moderated by the genotype of the 5‐HTTLPR, MAO-A, and DAT1 genes in young adults. The genotype of the DRD4 gene in young adults did not moderate this relationship (*P*=.41). In particular, high scores on FAD AR were positively associated with high levels of IA, but only in the presence of the L/x genotype of the 5‐HTTLPR gene (*B*=.37; *t_100_*=3.54, *P*<.001; S/S: *P*=.85; Figure 4).

**Figure 5:**
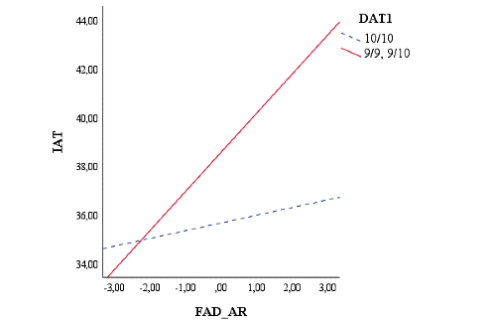
Moderation of youth’s DAT1 genotype on the relationship between the youth’s family functioning and IAT score. DAT: dopamine active transporter; FAD_AR: Family Assessment Device Affective Responsiveness; IAT: internet addiction test.

In addition, the 9/x genotype of the DAT1 was significantly related with high scores on IAT (*B*=0.42; *t_100_*=3.47, *P*<.001; 10/10: *P*=.56; Figure 5).

**Figure 6:**
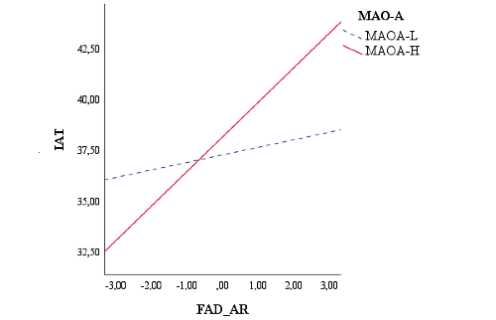
Moderation of the youth’s MAO-A genotype on the relationship between youth’s family functioning and IAT score. FAD_AR: Family Assessment Device Affective Responsiveness; IAT: internet addiction test; MAO-A: monoamine oxidase A; MAOA-H: MAO-A high-activity variant; MAOA-L: MAO-A low-activity variant.

Finally, the results showed a significant interactive effect of the MAOA-H polymorphisms (*B=*0.44; *t_100_*=3.55, *P*<.001; MAOA-L: *P*=.50; Figure 6).

## Discussion

### Principal Findings

This exploratory study aims to investigate the possible biological, psychological, and environmental risk factors associated with IA in a young adult population. On the basis of a biopsychosocial model [71] that considered IA as a result of a mutual influence between individual genotype, psychological profile, and social environment, this study aimed to verify the possible influence of genetic polymorphisms in MAO-A, 5HTTPR, DRD4, and DAT1 genes, considering the role played by the perception of young adults of their family functioning and their psychopathological difficulties. We considered the dopaminergic system (DAT and DRD4 genes), the serotonergic system (5-HTTPLR gene), and the monoamine metabolism pathway (MAO-A gene) for their central role in impulsive behaviors [92-94]. The neurotransmitter systems encoded by these genes are reportedly associated with alcoholism or other addictions [45,72,73,75,95-98]. Moreover, several studies have shown that these genetic polymorphisms contribute to the susceptibility of an individual to environmental influences [99-101] and can act as moderators in associations between the quality of the emotional environment provided by parents and the evolutionary outcomes of young adults [102].

Our first hypothesis was that the genotypes of the 5-HTTPR, DAT1, DRD4, and MAO-A genes differed significantly between the IA and non-IA groups. However, we found no significant association between IA and any genetic polymorphisms, in neither males nor females. Specifically, we found no association between the 5-HTTPR polymorphism and IA. Previous studies [36,41] have reported that the S/S genotype HTTLPR increased the risk of IA. Similarly, our study indicated a higher percentage of the S/S genotype in the IA group than in the control group, but there was no significant difference. Moreover, we found no association between the DAT1 genotype and IA. Previous studies have shown that the DAT1 polymorphism is associated with addiction behavior, such as alcoholism [103,104], substance use/abuse [105], and pathological gambling [106]. Some studies [72,107] have suggested that the DAT1 genotype 9/9 is associated with a higher risk for addictive disorders. However, the findings of our study are in line with the findings from studies by other researchers [74,75] that showed no significant association with internet-related addiction. Given these inconsistent findings, further research is needed to clarify whether the DAT1 gene may have an effect on IA. In addition, we found no differences in the DRD4 genotype between the IA and non-IA groups. The DRD4 4/4 polymorphism was shown to be a risk genotype for substance-related disorders [108]. Moreover, it was found to be correlated with decreased risk for inattention [34,35] and, consequently, with increased duration of internet use and a higher risk of IA [36]. However, other studies have underlined a higher risk for addictive disorders associated with the 7-repeat allele of DRD4 [109,110]. In our sample, we found a rarity of the 7-repeat allele (11/104, 10.5%) and, given that previous studies have shown that there was a statistically significant difference in allele distribution of the DRD4 gene between ethnicities [111], further evaluation is necessary for a better understanding of the possible influence of the DRD4 genotype on IA in specific ethnic groups. Finally, regarding the MAO-A gene, no significant association was found with IA. However, the literature has reported mixed findings with regard to its genotype that could be considered a higher risk to impulsive behaviors, with some studies showing a significant association with the MAOA low-activity variant [45,46], other studies with the MAOA high-activity variant [43,44], and some other studies that have not found any association [112,113].

Our second hypothesis was that the quality of family functioning perceived by youths and their psychological profiles differed significantly between the IA and non-IA groups. As expected, we found that youths in the IA group reported significantly higher scores (representative of a poorer family functioning) on the subscale of FAD AR and on all psychopathological areas considered (ie, depressive and anxiety symptoms and avoidant personality problems) than their peers in the control group. These findings are consistent with previous studies that have underlined that poor quality of family functioning is one of the main social-environmental risk factors for the onset and maintenance of addictive behaviors among young adult populations [51,52], including IA [49,50,114,115]. Families with poor AR are characterized by difficulties in showing their emotions [84]. In this field, some authors have suggested that, given that feelings are not expressed or tolerated, youths may be more susceptible to cope with their internal stress by themselves [116] and may use the internet excessively as a strategy to cope with negative emotions resulting from the interpersonal relationships with parents [117,118]. Moreover, it has been suggested that young adults who perceive a lack of supportive and intimate relationships with their parents are more likely to search for social support from virtual interactive experiences [119].

With regard to the possible association between IA and psychological difficulties of young adults, our results confirmed that youths of the IA groups showed higher scores on depressive, anxiety, and avoidant personality problems. These findings are in line with previous studies that have shown that young adults affected by IA also reported the presence of more severe psychopathological symptoms than their peers without IA [15], especially in the areas of depression [76,77], anxiety [120], and traits of avoidant personality disorder [70]. The nature of the association between IA and other symptomatic psychopathological areas is controversial. International scientific literature has suggested that psychopathological symptoms may cause or contribute to the onset of IA or, conversely, IA may cause or further exacerbate the course of other psychopathological symptoms [76,121,122]. However, recent longitudinal studies [123,124] have produced new evidence on the predictive role of psychological suffering on IA. In this field, some authors have suggested that youths may be at higher risk of developing IA in an attempt to cope with their psychological problems [2]. For example, a youth affected by depression could use the internet as a strategy to escape and cope with feelings of sadness and low self-esteem [122]; excessive use of the internet could be used by young people in an attempt to manage anticipatory anxiety associated with stressful situations and life events [66]; people with avoidant personalities are often socially inhibited and have difficulty communicating with others in face-to-face situations [125], and, thus, they may use web-based communication to seek interpersonal interactions in a way safer and easier for them [126].

Our third hypothesis was that the psychopathological symptoms of young adults might mediate the relationship between FAD AR and IA. International research has shown that poor quality of family functioning is a significant predictor of the psychopathological symptoms of young adults [2,127], which, in turn, has been reported as a significant predictor of IA [123,124], suggesting a possible mediation role. Our results showed that depressive symptoms and avoidant personality problems of young adults mediated the relationship between FAD AR and IA. Moreover, our results have confirmed previous studies on the significant positive association between the perception of a poor AR of families of young adults to their depressive problems [127,128] and avoidant personality traits [129]. However, anxiety symptoms in youths did not mediate the relationship between FAD AR and IA, although the direct effect on IA was confirmed and is in line with studies by Sepehrian and Lotf [130] and Razieh et al [131]. The results of multiple mediation analyses showed only a significant total indirect effect, indicating that depression and anxiety problems, and avoidant personality traits together significantly explained the association between youth’s family AR and IA. However, the specific effects of the 3 mediators were not significant. As evidenced by Hayes [91], these results are only apparently contradictory, and could be as a result of the presence of a correlation between the mediators and the small size of the sample. Although previous research has underlined that psychopathological symptoms of young adults mediate the relationships between environmental risk factors (eg, parental psychopathological symptoms, stressful life events) and IA [132,133], this is the first study to explore the possible role played by family functioning in these processes.

Finally, our fourth hypothesis was that genetic polymorphisms in MAO-A, 5HTTPR, DRD4, and DAT1 genes might moderate the relationship between family functioning and IA. Our results showed that the relationship between FAD AR and IA was moderated by the genotype of the 5‐HTTLPR, DAT1, and MAO-A genes in young adults. In particular, poor AR perceived by youths in their family functioning was associated with a high level of IA, but only in the presence of the L/x genotype of the 5‐HTTLPR gene, the MAOA-H genotype, and the 9/x genotype of the DAT1. These findings are in accordance with recent evidence from geneXenvironment (GxE) interaction studies, which have indicated that not all individuals are susceptible to environmental influences to the same extent [134]. In this regard, the individual genotype (genetic variations) is considered to play a crucial role in moderating individual sensitivity to environmental exposure [79] and to the development of psychopathology related to addiction [48]. In particular, although studies by Lee et al [41] and Sun et al [36] have reported a significant association between IA and the presence of 2 copies of the short allele (S/S) of the 5-HTTLPR, our results are in line with previous studies that have reported an interaction effect between the L allele with stressful life events and a poor quality of family relationship on the onset of psychopathological problems [109,135], including difficulties in the area of addiction [136,137]. Moreover, the presence of the MAOA-H genotype gene has been shown to be a significant moderator in the relationship between family environmental exposure and addiction-related problems [138,139], and individuals carrying the 9-repeat allele of the DAT1 gene are at higher risk of alcoholism [140] and other addictive problems [72,107]. However, to the best of our knowledge, this is the first study to explore the possible influence of GxE on IA.

### Possible Limitations, Strength, and Implications

This study has some limitations. First, the small sample size and the resulting limited statistical power that should be taken with caution in our preliminary findings, which should be confirmed by further studies with larger samples. Moreover, the cross-sectional nature of the study did not allow testing of causal links between the variables taken into account, which should be explored in subsequent longitudinal studies. Moreover, we used a self-report tool for the assessment of IA of young adults, their family functioning, and psychopathological difficulties. Although these tools have proven their worth in numerous studies, future studies should evaluate these variables through more robust methodologies, such as observational procedures or clinical interviews. Despite these limitations, this exploratory study is the first to focus on the possible moderation of genetic polymorphisms in MAO-A, 5-HTTPR, DRD4, and DAT1 genes on the relationship between the quality of family functioning and IA of young adults, reporting significant GxE effects. Overall, our findings add new evidence with regard to the biological, individual, and social risk factors associated with IA in the young adult population, which can be evaluated for the development of more targeted and effective intervention programs.

## Abbreviations

5-HT: Serotonin
5-HTTLPR: Serotonin-transporter linked polymorphic region
5-HTTPR: Serotonin-transporter
AR: affective responsiveness
ASR: Adult Self-Report
DA: dopamine
DAT: dopamine active transporter
DRD4: dopamine D4 receptor
FAD: Family Assessment Device
GxE: geneXenvironment
IA: internet addiction
IAT: internet addiction test
MAO-A: monoamine oxidase A
MAOA-H: MAO-A high-activity variant
MAOA-L: MAO-A low-activity variant
VNTR: variable number of tandem repetitions
